# A next-generation microarray further reveals stage-enriched gene expression pattern in the blood fluke *Schistosoma japonicum*

**DOI:** 10.1186/s13071-016-1947-x

**Published:** 2017-01-10

**Authors:** Pengfei Cai, Shuai Liu, Xianyu Piao, Nan Hou, Hong You, Donald P. McManus, Qijun Chen

**Affiliations:** 1 MOH Key Laboratory of Systems Biology of Pathogens, Institute of Pathogen Biology, Chinese Academy of Medical Sciences & Peking Union Medical College, Beijing, People’s Republic of China; 2 Molecular Parasitology Laboratory, QIMR Berghofer Medical Research Institute, Queensland, Australia; 3 Key Laboratory of Zoonosis, Shenyang Agriculture University, Shenyang, People’s Republic of China

**Keywords:** *Schistosoma japonicum*, Microarray, Gene profiling, Stage-enriched expression, Developmental biology

## Abstract

**Background:**

Schistosomiasis is caused by infection with blood flukes of the genus *Schistosoma*, and ranks, in terms of disability-adjusted life years (DALYs), as the third most important neglected tropical disease. Schistosomes have several discrete life stages involving dramatic morphological changes during their development, which require subtle gene expression modulations to complete the complex life-cycle.

**Results:**

In the current study, we employed a second generation schistosome DNA chip printed with the most comprehensive probe array for studying the *Schistosoma japonicum* transcriptome, to explore stage-associated gene expression in different developmental phases of *S. japonicum*. A total of 328, 95, 268 and 532 mRNA transcripts were enriched in cercariae, hepatic schistosomula, adult worms and eggs, respectively. In general, genes associated with transcriptional regulation, cell signalling and motor activity were readily expressed in cercariae; the expression of genes involved in neuronal activities, apoptosis and renewal was modestly upregulated in hepatic schistosomula; transcripts involved in egg production, nutrition metabolism and glycosylation were enriched in adult worms; while genes involved in cell division, microtubule-associated mobility, and host-parasite interplay were relatively highly expressed in eggs.

**Conclusions:**

The study further highlights the expressional features of stage-associated genes in schistosomes with high accuracy. The results provide a better perspective of the biological characteristics among different developmental stages, which may open new avenues for identification of novel vaccine candidates and the development of novel control interventions against schistosomiasis.

**Electronic supplementary material:**

The online version of this article (doi:10.1186/s13071-016-1947-x) contains supplementary material, which is available to authorized users.

## Background

Schistosomiasis, a debilitating and chronic disease caused by infection with blood flukes (digenetic trematodes) of the genus *Schistosoma*, remains one of the most significant parasitic diseases worldwide, afflicting more than 230 million people, with about 800 million exposed to the risk of the infection [1, 2]. Schistosomiasis caused about 3.31 million DALYs in 2010, exceeded only by intestinal nematode infections and leishmaniasis, in the list of global neglected tropical diseases [3]. *Schistosoma mansoni*, *S. haematobium* and *S. japonicum* are the three main species of clinical relevance. Currently, there are no practical anti-schistosome vaccines available. The repeated use of a single effective drug, praziquantel, is required for schistosomiasis treatment, while a variety of morbidity management strategies have been adopted for control of the disease [4, 5].

The schistosome life-cycle involves an aquatic snail as an intermediate host and a mammal as definitive host [6]. Schistosome cercariae are shed from infected snails under a light stimulus and are released into water resources. The free-swimming cercariae infect a mammalian host by skin contact. After skin penetration, the larvae lose their tails and transform into schistosomula. Once entering into capillaries or lymphatic vessels, they are carried to the heart and lungs within 3–5 days depending on the species. The lung-stage schistosomula continue migration to the hepatic portal system at about 14-days post-infection, where the juveniles pair up and become sexually mature. Then the schistosomes in copula migrate to the mesenteric veins (*S. mansoni* and *S. japonicum*) or the pelvic venous plexus (*S. haematobium*), where the female worms lay eggs intravascularly, with varied patency periods among the species. Some eggs are lodged in tissues causing disease whereas others enter the intestine or bladder and are excreted from the host. The mature eggs hatch under favourable conditions to release miracidia which penetrate a snail host and develop asexually into mother and then daughter sporocysts, within which cercariae are produced, which are then released from the snail and continue the life-cycle.

The availability of schistosome transcriptome [7, 8] and genome sequences [9–11] for the three major *Schistosoma* spp., provides an invaluable resource to profile gene expression across different developmental stages and between the sexes. In this respect, high-throughput technologies, such as microarrays [12–18], serial analysis of gene expression (SAGE) [19–21], digital gene expression (DGE) [22], and, more recently, RNAseq [23, 24] have been employed in the analysis of gene profiling in schistosomes. These pioneering investigations have provided unique information on developmental-enriched, gender-biased, tissue-specific, strain-specific and host-associated gene expression features within schistosomes [12, 14, 25–28], revealing critical insight on the biology of these parasites. With respect to using microarray platforms, the interpretation of microarray experiment depends on the quality of genetic information contained in the collection of DNA templates employed for probe design. The first-generation of schistosome cDNA chips were printed based on EST transcripts, so that the data obtained from these chip experiments resulted in a poor interpretation due to the problems in annotating these ESTs [12–14]. We considered it essential to generate a second generation DNA microarray with a well-curated probe design, based on both transcriptomic and genomic sequences, in order to increase our understanding of schistosome biology.

We have constructed a second generation schistosome DNA chip printed with the most comprehensive coverage of probes, designed based on *S. japonicum* and *S. mansoni* genomic and transcriptomic sequences for transcriptomic studies [29–31]. Here, we have identified stage-enriched transcripts in cercariae, hepatic schistosomula, adult worms and eggs using this next-generation DNA microarray. This study presents a comprehensive view of the expression features of stage-enriched genes for four developmental phases of *S. japonicum*, and provides novel insights on schistosome developmental biology.

## Methods

### Parasite materials

*Schistosoma japonicum*-infected snails (*Oncomelania hupensis*) were purchased from Hunan Institute of Parasitic Diseases, Yueyang, China. Cercariae were shed from these snails under light stimulation and were collected. Hepatic schistosomula at 14 days post-infection (p.i.) were perfused from *S. japonicum*-infected New Zealand rabbits *via* the vascular system. Mixed adult worms were perfused from *S. japonicum*-infected rabbits at 6 weeks p.i. Schistosome eggs were purified from liver tissues of infected rabbits (6 weeks p.i.) by enzyme digestion [32]. All parasite samples (except eggs) were soaked in RNAlater (Ambion, CA, USA), and stored at -80 °C until total RNA extraction. Total RNA from eggs was isolated immediately after purification.

### Total RNA isolation

Total RNA samples were isolated from *S. japonicum* cercariae, hepatic schistosomula, adult worms and eggs using RNeasy Mini kits (QIAGEN, GmbH, Hilden, Germany) according to the manufacturer’s instructions. Potential contaminating genomic DNA was removed from RNA samples using a Turbo DNA-free kit (Ambion, CA, USA). The quantity of RNA in each sample was assessed by a NanoDropND-1000 spectrophotometer (NanoDrop Technologies, Wilmington, DE). The integrity of total RNA in each sample was checked by denaturing agarose gel electrophoresis (Additional file 1: Figure S1).

### Microarray construction and hybridization and subsequent data analysis

A schistosome genome-wide microarray was employed for profiling the gene expression in *S. japonicum* cercariae, hepatic schistosomula, adult worms and eggs. The details regarding the design and construction of the microarray, the hybridization method, and feature extraction have been reported [29–33]. For each target sequence, 3 or 4 pairs of complementary oligonucleotide probes (forward and reverse, 60-mer) were designed (in total 145,000 probes). Probes with random sequences were printed as negative controls (background signal), while eight spike-RNA probes from the intergenic sequence of yeast were used as hybridization controls. Microarrays were printed in a 12 × 135 K feature format (Roche NimbleGen) representing 41,982 features. cDNA was labelled with a fluorescent dye (Cy3-dCTP) using a cRNA Amplification and Labelling Kit (CapitalBio, Beijing, China) [34]. Hybridization was performed using three biological replicates for all samples by CapitalBio, Beijing, China. Procedures for array hybridization, washing, scanning, and data acquisition were performed according to the NimbleGen Arrays User’s Guide. The arrays were scanned using a MS200 scanner (NimbleGen Systems) at 2-μm resolution, and NimbleScan software (NimbleGen) was employed to extract fluorescent intensity raw data from the scanned images. Normalized gene expression data were generated using the Robust Multichip Average (RMA) algorithm [35, 36]. Outlier probes were identified and their contribution was reduced at the reported gene expression level [36]. The expression value of a gene is a weighted average of all forward or reverse probe sets when both background correction and quantile normalization are performed.

### Bioinformatics analysis on stage-enriched mRNA and EST transcripts

mRNA and EST transcripts highly enriched in cercariae, hepatic schistosomula, adult worms and eggs of *S. japonicum* were retrieved from the NCBI database (http://www.ncbi.nlm.nih.gov/sites/batchentrez) based on fold-change (FC = the mean intensity/the median of the mean intensity values of the four developmental stages) values. (FC ≥ 2 for both forward and reverse probe sets, and three biological replicates were used for each stage). Student’s *t*-test was used to determine differentially expressed genes between one particular stage and any of the other three stages [28, 30] (*P* < 0.05). Heat maps were constructed based on the transformed log_2_FC values (forward probe sets) using HemI 1.0 software [37]. Blast2GO was used to annotate the four gene sets functionally [38]. A comprehensive re-annotation was performed against these gene sets using the BLASTx algorithm, with the annotation of *S. mansoni*, *S. haematobium* and *Clonorchis sinensis* homologues as a reference. For possible improved annotation, potential conserved protein domains were searched against genes annotated as hypothetical protein or unknown in the NCBI CDD database (v3.14) [39].

### Quantitative real-time PCR

A total of 20 stage-enriched genes were selected for validation using qRT-PCR as described [29]. One microgram total RNA samples were reverse transcribed into first-strand cDNA using a SuperScript III Reverse Transcriptase Kit (Invitrogen, Carlsbad, CA, USA) with oligo (dT) 15 primer. The cDNA products were diluted 20-fold with nuclease-free water before undertaking the qPCR. Each 25 μl PCR reaction contained 12.5 μl of 2 × Brilliant II SYBR Green QPCR Master Mix (Agilent, USA), 1 μl cDNA, 1 μl of the forward and reverse primer pair (Additional file 2: Table S1), and 10.5 μl of sterile water. PCR cycling conditions were as follows: 95 °C for 10 min, followed by 40 cycles of 30 s denaturation at 95 °C and 1 min annealing and extension at 60 °C. A dissociation step (95 °C for 15 s, 60 °C for 1 min, 95 °C for 15 s and 60 °C for 15 s) was performed to confirm the amplification specificity for each gene. 26S proteasome non-ATPase regulatory subunit 4 (*PSMD4*) [29, 40] was employed as a house-keeping gene in the assays. PCR reactions were performed in technical triplicates on the 7300 Real-Time PCR system (Applied Biosystems). The relative expression level of each gene was analysed using SDS 1.4 software (Applied Biosystems). Correlations between the microarray and qPCR results for 20 stage-enriched genes were analysed with the Spearman’s rho.

## Results and discussion

### Global view of stage-enriched mRNA transcripts in *S. japonicum*

By employing a microarray with the most comprehensive probe coverage design to date, signal intensities from 3571, 1014, 1728 and 3381 sequences were found to be enriched (FC of mean of intensity value to the median of the mean of intensity values of the four stages ≥ 2) in cercariae, hepatic schistosomula, adult worms and eggs, respectively. Based on the initial screening, we further retrieved a total of 1768 potential mRNA transcripts and 470 expressed sequence tags (ESTs) associated with developmental stages from the NCBI database (Additional file 3: Table S2). The gene collection was further filtered by requiring FC values from both forward and reverse probe sets ≥ 2. This filtration finally retained 328, 95, 268 and 532 mRNA transcripts highly enriched in cercariae, hepatic schistosomula, adult worms and eggs, respectively (Additional files 4, 5, 6 and 7: Tables S3–S6), which contrasted with 128, 31, 83 and 84 ESTs, respectively, highly enriched in these four stages (Additional files 8, 9, 10 and 11: Tables S7–S10). However, the percentage of genes that were annotated as hypothetical protein or unknown (23.57% in the mRNA data in contrast to 69.01% in the EST data), highlights the utility of the second generation *S. japonicum* DNA chip in profiling gene expression in this parasite.

**Fig. 1:**
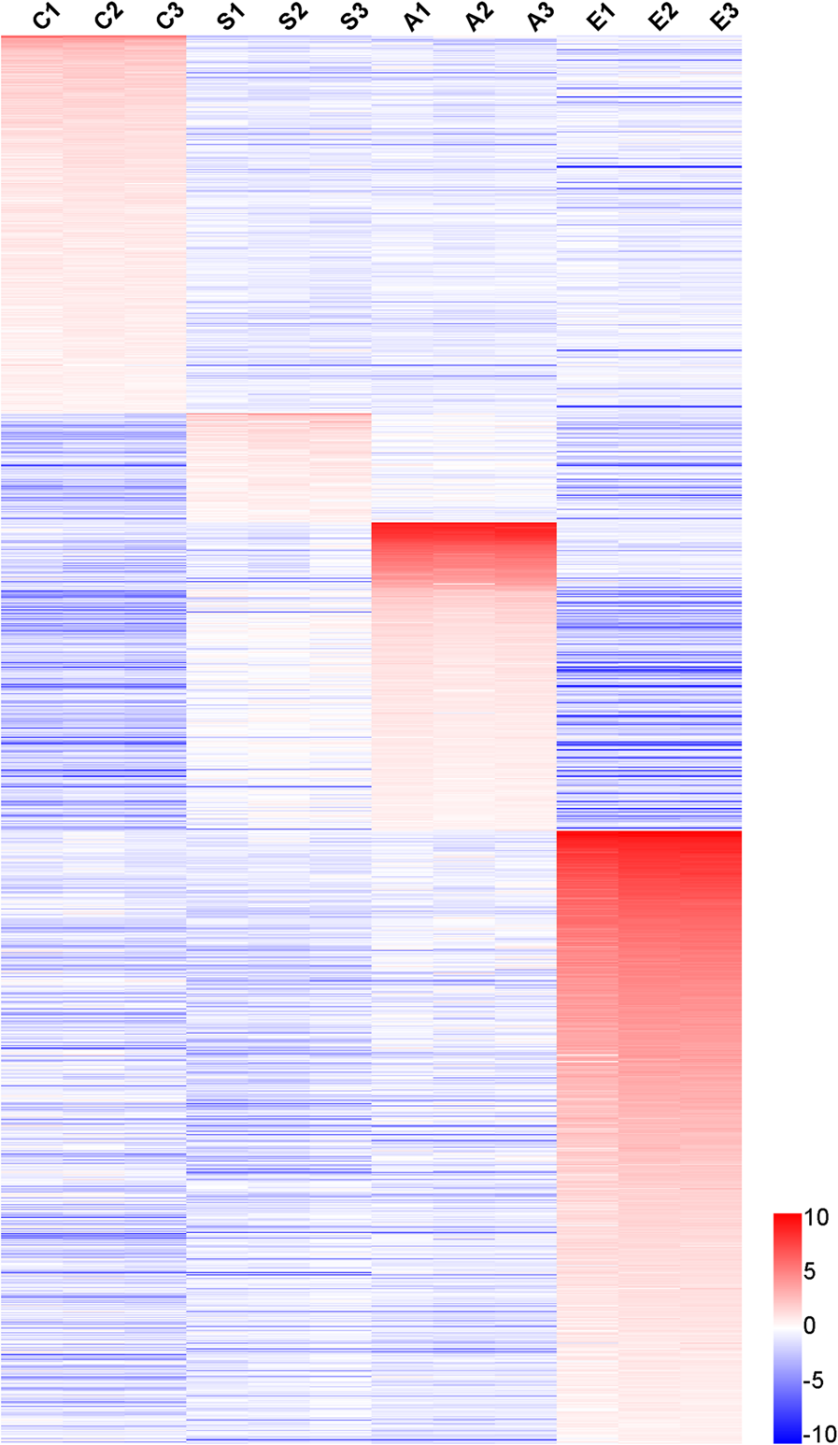
Heatmap for mRNA transcripts enriched in cercariae, hepatic schistosomula, adult worms and eggs. A total of 328, 95, 268 and 532 mRNA transcripts were enriched in cercariae, hepatic schistosomula, adult worms and eggs, respectively. The heatmap was created by HemI 1.0 based on the transformed data of log_2_ FC values. The data are based on the mean of weighted signal intensity values of forward probe sets (three biological replicates)

**Fig. 2:**
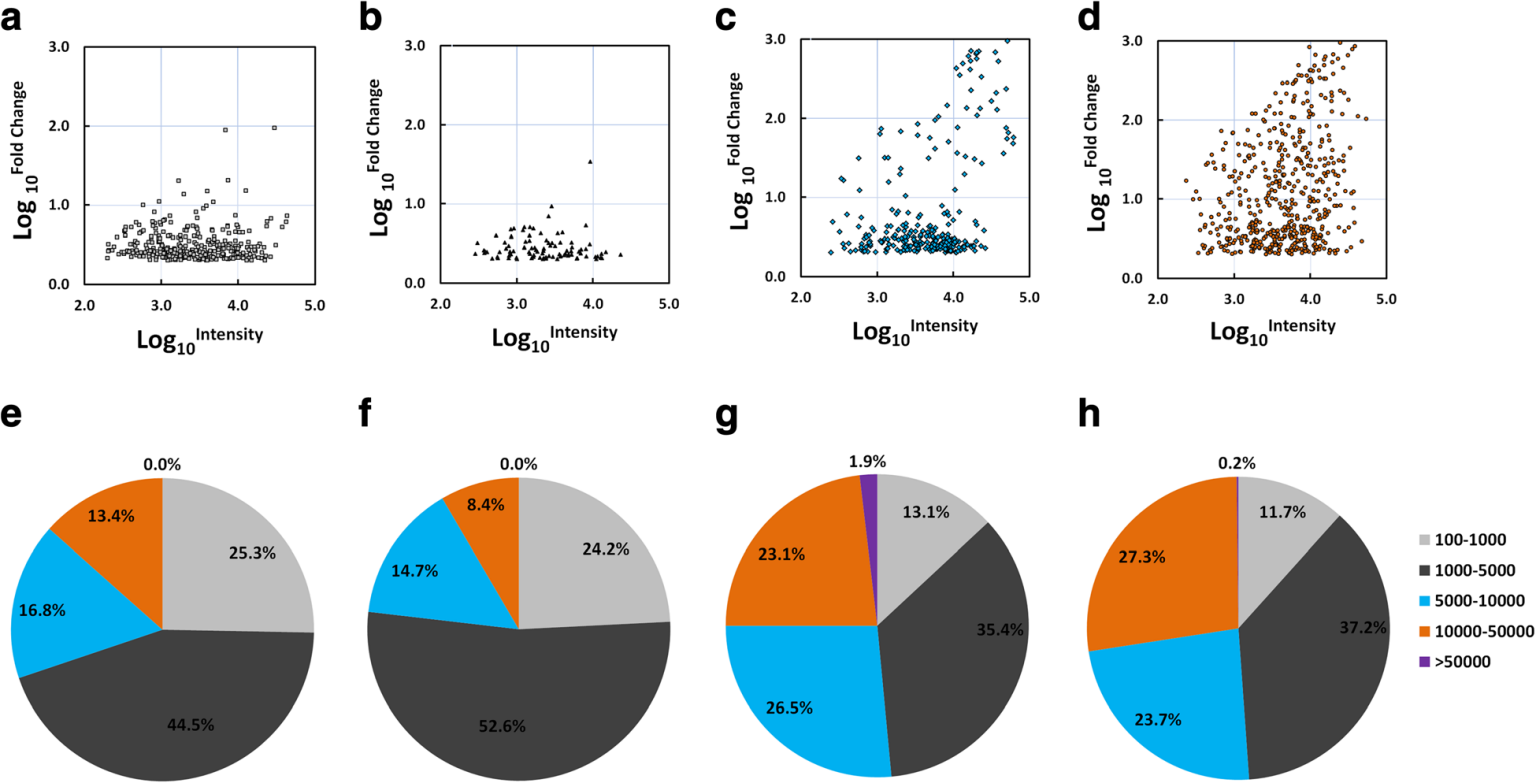
Bias ratio and signal intensity analysis of stage-associated genes. Scatter plot showing the distribution of the bias ratio and fluorescence intensity for mRNA transcripts enriched in cercariae (**a**), hepatic schistosomula (**b**), adult worms (**c**), and eggs (**d**). The y-axis corresponds to bias ratios (FC value) and the x-axis corresponds to the fluorescence intensities, both of which are log_10_-transformed. Pie diagrams representing the percentage of stage-enriched mRNA transcripts in cercariae (**e**), hepatic schistosomula (**f**), adult worms (**g**), and eggs (**h**) showed by different fluorescence intensities

We observed that more mRNA transcripts were enriched in the egg stage than in the other stages, with a stronger biased expression (higher FC value) (Figs 1 and 2a-d). For example, 46.1% egg-enriched mRNA transcripts showed a strong biased expression (FC > 10); this number decreased to 22.0% in adult worms, and further dropped to only 3.0 and 1.1% in cercariae and hepatic schistosomula, respectively. A similar tendency was observed when analysing the stage-enriched EST transcripts (Additional file 12: Figure S2). In regards to fluorescence intensity, 13.4, 8.42, 25.0 and 27.5% mRNA transcripts enriched in cercariae, hepatic schistosomula, adult worms and eggs, respectively showed an average intensity value > 10,000 (Fig. 2e-h).

### Comparing the results with previous transcriptome data

A complete and accurate comparison of the results obtained in the current study with data from previous reports is hindered due to the following reasons. Firstly, the annotation of stage-enriched genes was not ideal in previous reports due to the fact that EST sequences were used for probe design coupled with less sequence homology information from other trematode species being available. Secondly, the annotation for the same gene may not have been unique. Thirdly, the screening criteria for stage-enriched genes may have varied among different studies. Nevertheless, we compared our data with these from previous *Schistosoma* transcriptome data [7, 13, 14, 23, 28] by manual checking. Globally, about 4.57, 10.07 and 12.97% genes enriched in cercariae, adult worms and eggs, respectively, were reported in previous studies (Additional files 4, 5 and 7: Tables S3, S4, S6). With respect to hepatic schistosomula (14 days p.i.), to our knowledge the only other relevant investigation on this particular stage was carried out on *S. mansoni* by Fitzpatrick et al. [28], but no enriched gene clustering was evident in that study. This was probably due to the fact a large number (15) of distinct stages were analysed [28], and this has made comparison with our data for hepatic schistosomula difficult.

### qPCR validation of the expression pattern of stage-enriched genes

**Fig. 3:**
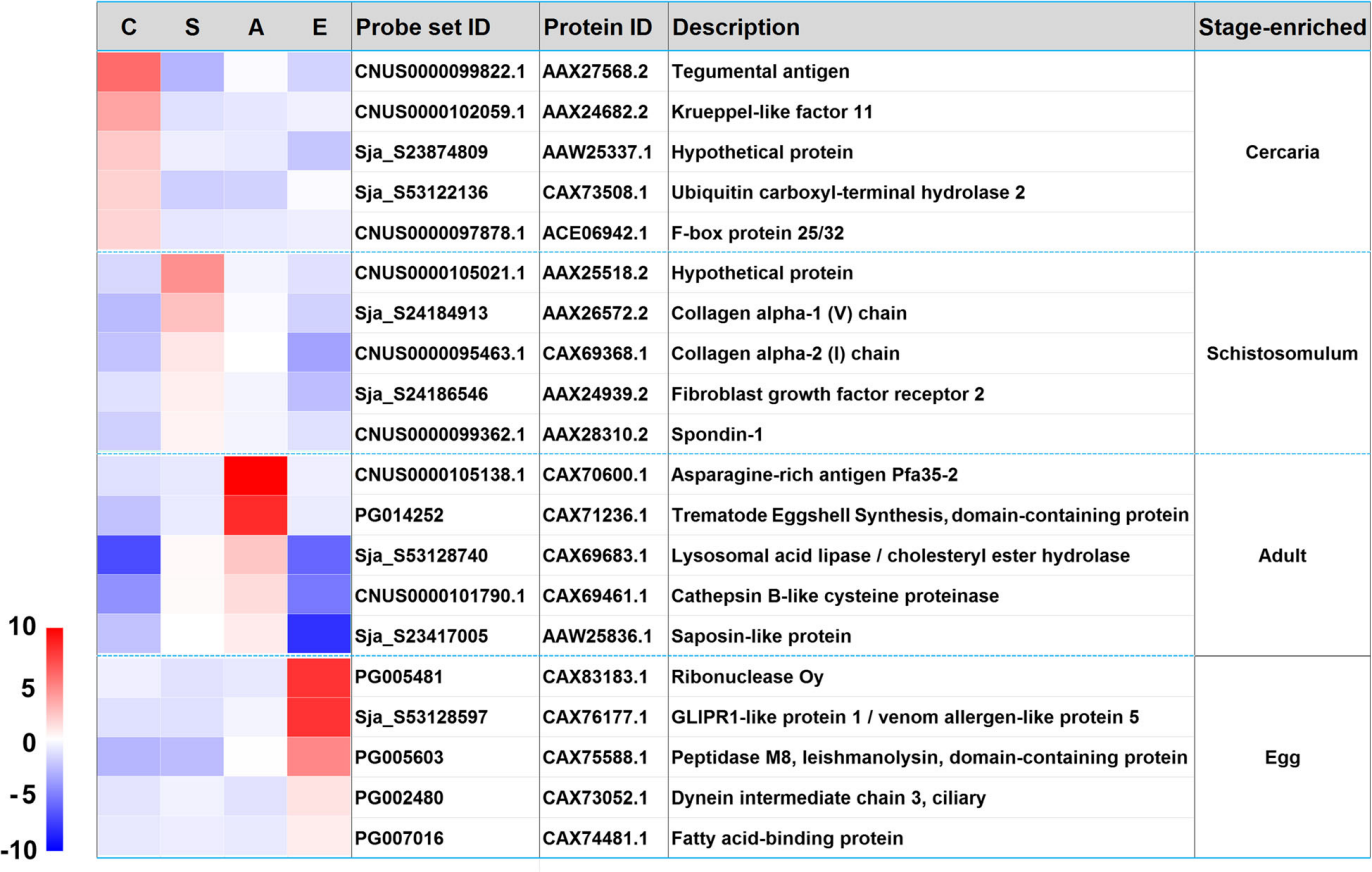
Twenty stage-enriched genes selected for qPCR validation. The heat map illustrates the hierarchical clustering of 20 stage-enriched genes based on the transformed data of log_2_ FC value of the three biological replicates. *Abbreviations*: C, cercariae; S, hepatic schistosomula; A, adult worms; E, eggs

**Fig. 4:**
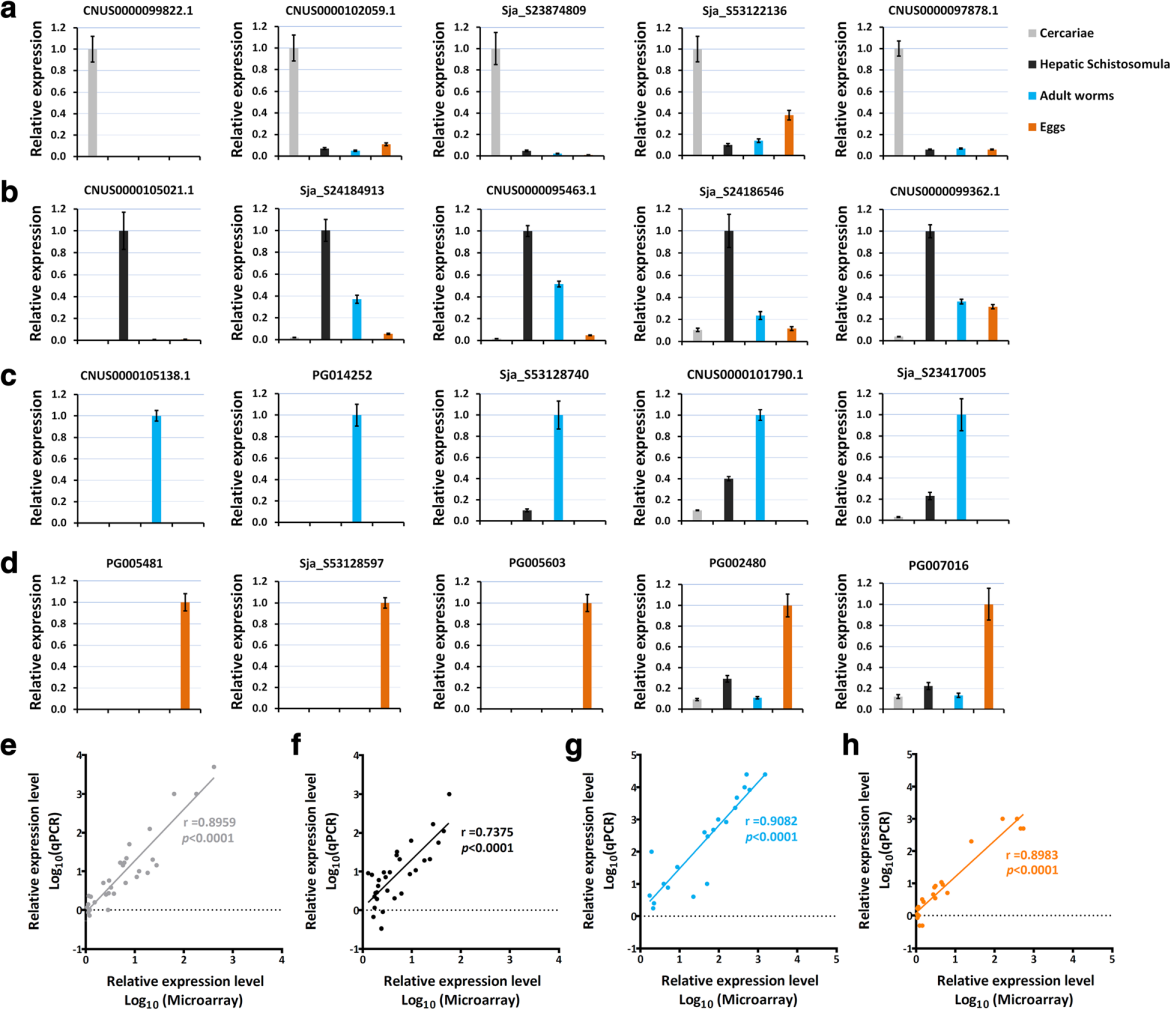
qPCR validation of stage-enriched genes. The expression of 5 selected genes enriched in cercariae (**a**), hepatic schistosomula (**b**), adult worms (**c**), and eggs (**d**), respectively, was quantified by qRT-PCR analysis. The *PSMD4* gene was used for internal normalization among the four developmental stages. The highest expression level in one particular stage was set as 1. The error bars represent the standard deviation for three technical replicates. Correlations between the microarray and qPCR results for the selected genes enriched in cercariae (**e**), hepatic schistosomula (**f**), adult worms (**g**), and eggs (**h**), were analysed using Spearman’s rho

A subset of 20 representative stage-enriched genes was selected for qRT-PCR validation (Figs 3 and 4a-d). Most genes were associated with important biological functions in each of the parasite forms. The expression of these genes at the four developmental stages validated by qRT-PCR analysis significantly correlated with the results obtained by microarray: for cercariae-enriched genes selected, *r*_(30)_ = 0.8959, *P* < 0.0001 (Fig. 4e); for hepatic schistosomula-enriched genes selected, *r*_(30)_ = 0.7375, *P* < 0.0001 (Fig. 4f); for adult-enriched genes selected, *r*_(20)_ = 0.9082, *P* < 0.0001 (Fig. 4g); for egg-enriched genes selected, *r*_(21)_ = 0.8983, *P* < 0.0001 (Fig. 4h).

### Putative functions predicted by GO analysis

**Fig. 5:**
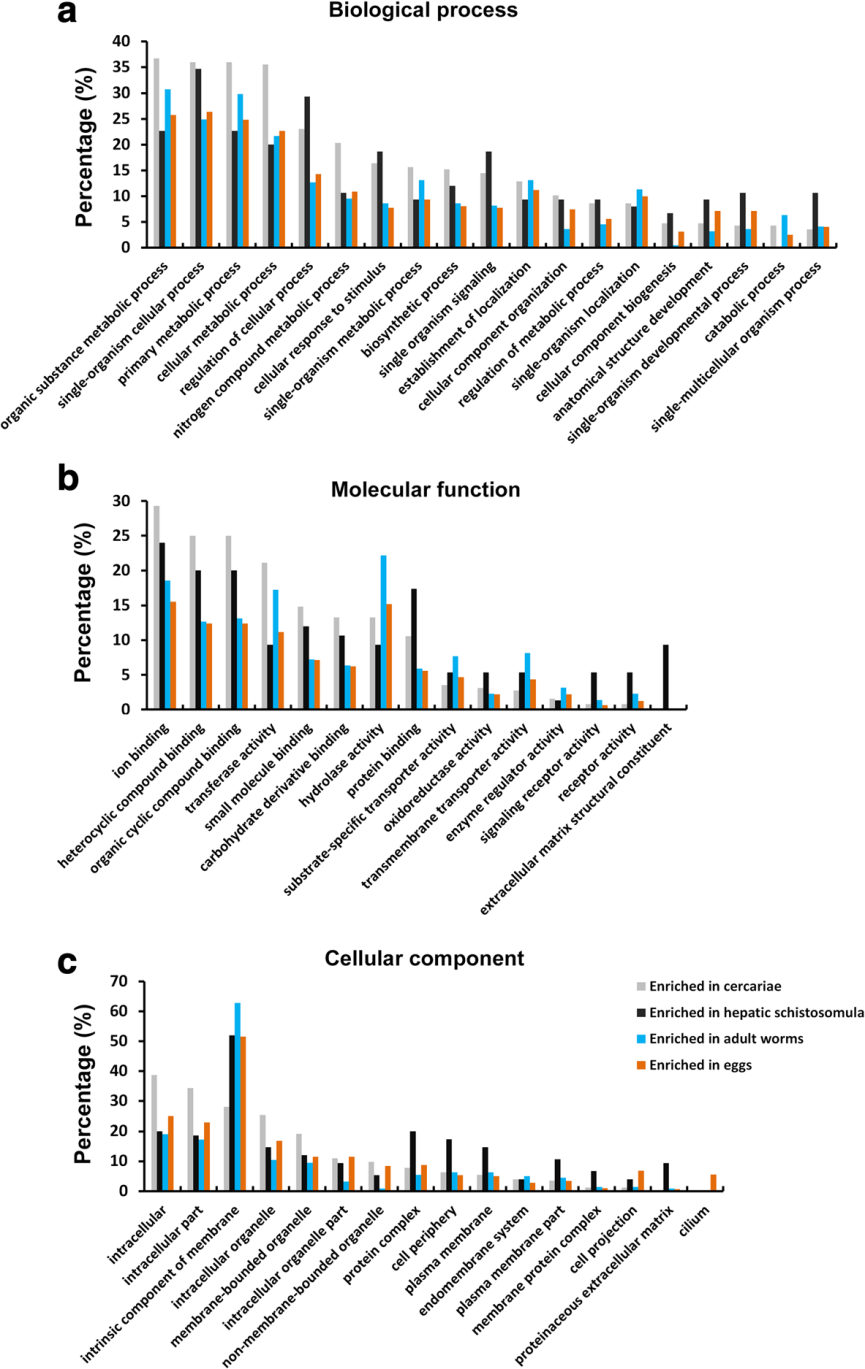
GO analysis of mRNA transcripts enriched in the four developmental stages of *S. japonicum*. The Blast2Go program defined the GO terms into three categories: biological processes (**a**), molecular functions (**b**) and cellular component (**c**). The y-axis shows the ratio of the number of mapped genes *versus* total number of genes in each cognate stage identified as a function of all available GO terms. The x-axis shows GO terms at the 3rd level

We analysed the potential biological functions of the stage-enriched genes in *S. japonicum* using GO classification [41] (Fig. 5, Additional files 13, 14, 15 and 16: Tables S11–S14). Of the biological process categories, the most highly enriched GO terms were organic substance metabolic process, single-organism cellular process, primary metabolic process and cellular metabolic process for cercariae, adult worms and eggs; the first three of these GO terms and regulation of cellular process were the most highly enriched GO terms for hepatic schistosomula. The percentages of genes involved in regulation of cellular process, cellular response to stimulus, and single organism signaling were higher in cercariae and schistosomula than those in adults and eggs. Of the molecular function categories, the percentages of genes involved in ion, heterocyclic compound and organic cyclic compound, small molecule and carbohydrate derivative binding were higher in cercariae and schistosomula than in adults and eggs. A higher percentage of genes related to protein binding, signaling receptor activity and receptor activity were observed in schistosomula, while the GO term extracellular matrix structural constituent was only evident for this stage. In addition, a higher percentage of genes involved in hydrolase activity were assigned to adult worms. In the cellular component categories, gene products localised to intracellular, intracellular part and intracellular organelle were more abundant in cercariae, while gene products localised to intrinsic component of membrane were more enriched in the other three stages. Further, genes with GO terms of protein complex, cell periphery, plasma membrane, plasma membrane part and proteinaceous extracellular matrix were relatively enriched in hepatic schistosomula. In addition, the GO term cilium was present only in the egg stage.

### The top 25 genes enriched in *S. japonicum* cercariae, hepatic schistosomula, adult worms and eggs

The top 25 highly stage-associated genes in cercariae, hepatic schistosomula, adult worms and eggs were analysed (Table 1). Collectively, the upregulated expression of these genes in cercariae indicates that signal transduction (ribosomal protein S6 kinase beta-2 [42]), vesicular trafficking (calcium-dependent secretion activator [43] and small GTPase Rab-protein 11 [44]) and energy metabolism (AMP deaminase [45] and 5′-AMP-activated protein kinase [46]) and transcriptional regulation (krueppel-like factor 11, homeobox protein SMOX-1, and retinoid X receptor RXR-2) are active processes in this stage.

**Table 1:** The top 25 genes enriched in *S. japonicum* cercariae, hepatic schistosomula, mixed adult worms and eggs

NCBI Nucleotide	NCBI Protein	Annotation	FC
Enriched in cercariae			
AY811679.1	AAX27568.2	Tegumental antigen	94.004
AY812964.1	AAW24696.1	Lysophosphatidic acid phosphatase type 6	89.015
AY808793.1	AAX24682.2	Krueppel-like factor 11	20.463
AY814888.1	AAP06195.1	Hypothetical protein	20.323
AY915869.1	AAX31090.1	UPF0506 domain containing protein	15.144
AY811006.1	AAX26895.2	Putative sodium-dependent transporter	14.884
FN319257.1	CAX74986.1	Ribosomal protein S6 kinase beta-2	13.668
AY813254.1	CAX83692.1	Gag-Pol polyprotein	11.090
AY812158.1	AAX28047.2	Calcium-dependent secretion activator	10.898
FN327240.1	CAX82964.1	UPF0364 protein	10.005
FN319112.1	CAX74840.1	Anti-inflammatory protein 16	9.750
AY809199.1	AAX25088.2	Dynein light intermediate chain 1 cytosolic	9.060
AY815066.1	AAW26798.1	Calpain	8.200
FN314407.1	CAX70140.1	Rab-protein 11	8.118
AY813232.1	AAW24964.1	DM9 domain-containing protein	7.327
AY915497.1	AAX30718.2	Homeobox protein SMOX-1	7.320
AY813605	AAW25337.1	Hypothetical protein	7.234
FN319705.1	CAX75429.1	THO complex subunit 1	6.827
AY813585.1	AAW25317.1	Hypothetical protein	6.756
AY811834.1	AAX27723.2	AMP deaminase	6.524
AY813088.1	AAW24820.1	Hypothetical protein	6.357
FN314484	CAX70217.1	Hypothetical protein	6.196
AY811464.1	ABA40369.1	5′-AMP-activated protein kinase subunit gamma-1	6.165
EU046089.1	AAW25910.1	Cercarial stage-specific protein Sj20H8	6.075
AY808884.1	AF129816_1	Retinoid X receptor RXR-2	6.011
Enriched in hepatic schistosomula			
AY809629.1	AAX25518.2	Hypothetical protein	33.897
AY810683	AAX26572.2	Putative collagen alpha-1(V) chain precursor	9.200
AY815366.1	AAW27592.1	Alpha-ketoglutarate-dependent dioxygenase alkB 6	6.931
AY813429.1	AAW25161.1	Hypothetical protein	5.290
AY810949.1	AAX26838.2	Homeobox protein engrailed-like SMOX-2	5.057
EZ000055.1	ACE06835.1	Vacuolar protein sorting-associated protein 29	5.021
AY810397.1	AAX26286.2	Protocadherin Fat 4	4.839
AY811075.1	AAX26964.2	Hypothetical protein	4.831
AY815532.1	AAW27264.1	Hypothetical protein	4.727
AY814356	AAW26088.1	RhoGAP domain containing protein	4.610
AY811025.1	AAX26914.2	Serine/threonine-protein kinase Sgk1	4.342
AY809477.1	AAX25366.2	SAM and SH3 domain-containing protein 1	4.248
FN314446.1	CAX70179.1	Annexin A3 (Annexin III)	4.037
AY814048.1	AAW25780.1	Basic proline-rich protein-like isoform	3.967
AY808501.1	AAR28090.2	Nuclear receptor subfamily 4 group A	3.956
AY809584.1	AAX25473.2	Hypothetical protein	3.894
AY812287.1	AAX28176.2	Run domain Beclin-1 interacting and cysteine-rich containing protein	3.806
AY813648.1	AAW25380.1	Hypothetical protein	3.439
AY915540.1	ABA40872.1	Leishmanolysin-like peptidase	3.419
AY812557.1	AAX28446.2	Aromatic-L-amino-acid decarboxylase	3.335
AY808377.1	AAX24266.2	Regulator of G-protein signaling 3	3.250
FN313634.1	CAX69368.1	Collagen alpha-2(I) chain	3.244
AY813683.1	AAW25415.1	Delphilin	3.240
AY812144.1	AAX28033.2	Hypothetical protein	3.212
AY813563	AAW25295.1	Hypothetical protein	3.203
Enriched in mixed adult worms			
FN314868.1	CAX70600.1	Asparagine-rich antigen Pfa35-2	1651.245
EZ000096	ACE06876.1	Putative eggshell protein precursor	934.084
FN314999	CAX70731.1	TES domain containing protein	704.455
AY813556.1	AAW25288.1	Hypothetical protein	692.180
AY814029	AAW25761.2	Stress protein DDR48 (DNA damage-responsive protein 48)	678.514
FN313935.1	CAX69669.1	Stress protein DDR48 (DNA damage-responsive protein 48)	665.581
FN317103	CAX72834.1	Stress protein DDR48 (DNA damage-responsive protein 48)	645.627
FN313912	CAX69646.1	TES domain containing protein	604.574
FN313715.1	CAX69449.1	TES domain containing protein	561.444
AY812810.1	AAW24542.1	Histidine-rich glycoprotein precursor	526.698
FN315504.1	CAX71236.1	TES domain containing protein	517.929
AY815518	AAW27250.1	TES domain containing protein	489.519
FN314997	CAX70729.1	TES domain containing protein	422.784
AY813405	AAW25137.1	TES domain containing protein	407.588
AY815264.1	AAW26996.1	Tyrosinase 1	346.094
AY812315.1	AAX28204.2	Hypothetical protein	330.410
FN330801	CAX83018.1	Stress protein DDR48 (DNA damage-responsive protein 48)	235.455
AY814142.1	AAW25874.1	Putative FAM75 family member	224.325
AY812904	AAW24636.1	Tyrosinase 2	209.523
FN315510.1	CAX71242.1	Hypothetical protein	164.941
AY814814	AAW26546.1	Cadherin	145.264
AY815418	AAW27150.1	Female-specific protein 800	135.097
FN316955	CAX72686.1	Prostatic spermine-binding protein precursor	132.448
AY222885	AAP05897.1	Stress protein DDR48 (DNA damage-responsive protein 48)	127.238
FN314903.1	CAX70635.1	Hypothetical protein	107.908
Enriched in eggs			
FN317800	CAX73529.1	Glutenin high molecular weight subunit DX5	1794.846
FN319280	CAX75008.1	Tetraspanin 22	1769.270
FN322023.1	CAX77751.1	Histidine-rich glycoprotein	1656.913
FN324495.1	CAX80219.1	Hypothetical protein	1549.720
FN326817	CAX82541.1	Histidine-rich glycoprotein	1523.735
FN317759.1	CAX73488.1	Similar to venom allergen-like (VAL) 25 protein	1062.695
FN324480.1	CAX80126.1	Hypothetical protein	938.553
FN321785	CAX77509.1	Ribonuclease T2	850.487
FN321171.1	CAX76897.1	Hypothetical protein	831.194
FN324498.1	CAX80222.1	Hypothetical protein	776.801
FN319117.1	CAX74843.1	CIA30 domain containing protein	665.147
FN317754	CAX73483.1	Tetraspanin	663.055
FN322724.1	CAX78439.1	Peptidase inhibitor 16	651.579
FN319142	CAX74870.1	Hypothetical protein	628.202
FN320551	CAX76277.1	Egg protein CP1531	592.491
FN326664	CAX82388.1	Hypothetical protein	577.505
AY816014.1	AAW27746.1	Ribonuclease S-4	534.668
FN321764.1	CAX77484.1	Cell wall integrity and stress response component 1	488.342
FN326758	CAX82480.1	Hypothetical protein	484.608
FN317167	CAX72898.1	Hypothetical protein	481.352
FN319216.1	CAX74944.1	Hypothetical protein	453.890
FN320451	CAX76177.1	GLIPR1-like protein 1/venom allergen-like protein 5	422.820
FN317231	CAX72962.1	GLIPR1-like protein 1/venom allergen-like protein 5	417.438
FN326877	CAX82601.1	Hypothetical protein	416.455
FN330952.1	CAX83183.1	Ribonuclease Oy	414.347

The over-expression of the top 25 genes in hepatic schistosomula appears to reflect a diversity of physiological activities, including transcriptional (homeobox protein engrailed-like SMOX-2 [47, 48], serum and glucocorticoid-regulated kinase 1 (SGK1) [49] and nuclear receptor subfamily 4 group A [50, 51]) and neuronal (protocadherin FAT4 [52], Aromatic-L-amino-acid decarboxylase [53] and delphilin [54]) activities, together with tegumental integrity (annexin A3 [55, 56]), skeletal morphogenesis (protocadherin FAT4 [57]) and endosome-to-Golgi retrieval (vacuolar protein sorting-associated protein 29 [58]).

In mixed adult worms, genes encoding a number of trematode eggshell synthesis (TES) domain-containing proteins, DDR48 stress proteins, an asparagine-rich antigen Pfa35-2, two distinct tyrosinase homologues, cadherin, female-specific protein 800 and a prostatic spermine-binding protein are listed in the top 25 enriched mRNA transcripts (Table 1). Most of these genes are female-biased expressed genes [59] with potential molecular functions in egg production [60].

In the egg stage, genes encoding a glutenin high molecular weight subunit DX5, egg protein CP1531, two histidine-rich glycoproteins, three ribonucleases, two tetraspanins, three venom allergen-like (VAL) proteins and cell wall integrity and stress response component 1 are present in the top 25 upregulated mRNA transcripts (Table 1). Notably, it has been shown that T2 ribonuclease omega-1 in soluble egg antigen is a major Th2 polarizing component, which is capable of regulating inflammasome activity [61]. It has been shown previously that VAL-5 is mainly present in the egg, miracidium and sporocyst developmental stages [62].

### Genes enriched in cercariae

Interestingly, a group of genes encoding transcription factors, i.e. homeobox protein SMOX-1 (AY915497), bhlhzip transcription factor max/bigmax (FN314500), pre-B-cell leukemia transcription factor 2 (AY809282), transcription factor 25 (AY808969), 20 (AY813668), BTF3 (EZ000130), TFIID subunit 3 (AY812404) and 7 (FN317813), IIIB subunit (AY812330), LIM/homeobox protein (AY915618) and transcriptional repressor NF-X1(AY813973) were actively transcribed in cercariae (Additional file 4: Table S3), indicating gene transcription may not be as silent as previously suggested in this stage. It has been shown that the highest ratio of miRNAs, the critical post-transcriptional regulators, in the total small RNA population was observed in cercariae compared with other different developmental stages of *S. japonicum* [32, 63], leading us to hypothesise that a specific group of genes may be actively transcribed in this aquatic stage. In addition, miRNAs may inhibit the translation of a subset of these transcripts, forming a repertoire of genes that make schistosomula ready to adapt to subsequent intra-mammalian life. Further, there is epigenetic control of gene expression in *S. mansoni* cercariae [64]. Overall, these observations indicate that active transcriptional regulation occurs at different layers in cercariae to subtly control gene expression in this stage.

We also observed that an extensive gene panel involved in cellular signalling transduction, i.e. F-box protein 25/32 (EZ000162), dual specificity mitogen-activated protein kinase 2 (AY815572), Serine/threonine kinase NLK (FN317434), Rho GTPase-activating protein 39 (FN317833), GDP/GTP exchange factor Sec2p domain containing protein (FN317362), Rho-associated protein kinase 1 (FN330915), mitogen-activated protein kinase 3 (EZ000180), Ran binding protein 9-related protein (AY812647), GTP-binding protein 2 (FN317377), NF-kappa-B inhibitor-interacting Ras-like protein 1 (AY812481), son of sevenless (AY915633), MAP kinase (AY594257), C-Jun-amino-terminal kinase-interacting protein 4 (AY808598), and regulator of G-protein signaling 7 (AY810841), were over-expressed in cercariae (Additional file 4: Table S3). These results support recent finding that three signaling pathways, extracellular signal-regulated kinase (ERK), p38 mitogen-activated protein kinase (MAPK), and protein kinase C (PKC), are modulated in cercariae in response to light and temperature cues as well as the skin fatty acid linoleic acid (LA) and are important in host penetration mechanisms [65].

In line with, and expanding on, previous transcriptional studies on schistosomes [13, 14, 66], genes encoding an array of cytoskeleton motor proteins, including dynein light intermediate chain 1, cytosolic (AY809199), troponins (FN317001 and AY809606), tensin-1 (AY809674), villin (AY808977), myosin light chain kinase, actin-related protein 5 (FN326677), dynamin (AY809889), catenin beta (AY814842), coronin (AY814365), dynein light chain Tctex-type 1 (AY811669) and alpha-actinin (FN326862) (Additional file 4: Table S3) were more highly expressed in cercariae than the other stages evaluated. Transcripts encoding LIM or PDZ domain-containing proteins, which contribute to cytoskeletal organisation, such as LIM/homeobox protein (AY915618), actin binding LIM protein 1 (AY813306), four and a half LIM domains protein 2 (FN317368), and PDZ and LIM domain protein 7 (FN317962) (Additional file 4: Table S3), were also enriched in cercariae. Proteomic studies also revealed that cytoskeleton-related proteins are abundant in schistosome cercariae [67]. Together, these data indicate modulated signalling and motor activities and rigid transcriptional regulation are the most important biological events in cercariae, which enable them to seek, invade and adapt to a suitable definitive host.

### Genes enriched in hepatic schistosomula

On invading a mammalian host, schistosomes have evolved several mechanisms to adapt to, and survive in, the hostile host environment; in particular, they develop a unique syncytial tegument, as well as mechanisms of antigenic mimicry [33], immune modulation [68] and evasion [69, 70]. In this study, we found extracellular matrix constituents, that are located in the tegumental protein assemblage, were enriched in hepatic schistosomula. These collagen components included, for example, collagen alpha-1(V) chain (AY810683, AY811988, and AY815998), alpha-1(IV) chain (AY809845), alpha-1(XXIV) chain (AY814344), alpha-2(I) chain (AY810097, FN313634) and alpha-2(V) chain (AY813923) (Additional file 5: Table S4). This observation raises the possibility that collagen components may form a protective barrier on the worm surface, which may help the schistosomula evade host attack.

Schistosomula undertake a lengthy migration in the mammalian host to the portal venous system, where they mature into adult worms and pair. This migration is closely associated with locomotion activity controlled by the neuronal system. The data presented here show that neuronal activities may be particularly active in hepatic schistosomula, which could be linked to the fact that responses to environmental cues from the host and the subsequent control of mobility are required to guarantee that they reach their destination [22]. A cohort of genes involved in neuronal activities in this stage includes netrin receptor unc5B (AY915275), nephrin (AY809045), caskin 2 (AY812623), spondin-1 (AY812421), as well as the previously described genes protocadherin FAT4, aromatic-L-amino-acid decarboxylase and delphilin. Although the precise functions of these genes in schistosomes remain unknown, there is evidence from other studies that at least three are involved in axon guidance. In mammals, it has been shown that the unc5B receptor, interacting with netrin-1, activates the downstream signal transduction pathway that mediates axon guidance [71]. A caskin ortholog in *Drosophila* is a cytoplasmic adaptor protein, which has been shown to mediate Lar signal transduction motor axon guidance [72]. Similarly, spondin-1 is an extracellular matrix protein, and previous research showed that its *C. elegans* ortholog functions in axon guidance and fasciculation in motoneurons [73]. Also, the expression of nephrin homologues has been observed in the central nervous system of mammals, and nephrin may potentially interact with glutamate receptors [74, 75].

In multicellular organisms, apoptosis is a highly controlled cellular process of programmed cell death which plays a key role in maintaining cell populations during an organism’s life-cycle. The apoptosis pathway has been suggested as a potential intervention target in schistosomes [76]. The activities of two central proteolytic enzymes involved in the apoptosis process, caspase-3 and -7, were shown to peak in *S. japonicum* schistosomula (14 days p.i.) [77]. The upregulated expression of caspase 7 (AY813428) in hepatic schistosomula was confirmed in this study (Additional file 5: Table S4). It is of note that a cohort of planarian neoblast-like cells with self-renewal function has been identified in *S. mansoni*, with a potential role in renewal of the tegument [78]. In this respect, fibroblast growth factor receptor 2, a crucial gene for the maintenance of neoblast-like cell population in schistosomes [79], was enriched in hepatic schistosomula (Additional file 5: Table S4), emphasising the requirement for rapid tegumental renewal during this period of fast-growth.

### Genes enriched in adult worms

One of the major biological roles of adult worms is to produce a large number of eggs, a key process in the schistosome life-cycle. As earlier mentioned, within the top 25 adult-enriched genes, most are associated with egg production. However, two pre-requisites for egg production are mating and nutrient acquisition. In fulfilment of the former process, the gene encoding gynecophoral canal protein has been shown upregulated in adults, with a dramatic bias towards male worms [59]. In regards to nutrient uptake, and consistent with a previous study [18], over-expression of a number of ‘blood processing’ proteases in adult worms was also revealed here. For instance, cathepsin family members, i.e. cathepsin C (FN315267), cathepsin D2-like (AY812817), cathepsin B-like (AY814095), cathepsin L (FN313884) and cathepsin L-like isoforms (AY222874, FN314782, and FN314778), and aminopeptidase N (FN317672) were readily identified as adult worm-enriched genes (Additional file 6: Table S5). In addition, saposin B domain-containing proteins (FN314931, FN315898 and FN314355), which have been proposed as being involved in nutrient acquisition by disrupting the membrane of red blood cells to release haemoglobin [80], were highly expressed in adult worms.

In schistosomes, glycosylation is a complex process which plays a crucial role in their biology, particularly in terms of immune modulation [81]. A subset of transcripts involved glycosylation in was enriched in adult worms of *S. japonicum*. These genes included beta-1,4-galactosyltransferase 4 (AY813412), glycosyltransferase 1 domain-containing protein 1 (FN319898), GDP-fucose protein O-fucosyltransferase 2 (AY810860), beta-1,3-galactosyltransferase 5 (AY814132), glycoprotein-N-acetylgalactosamine 3-beta-galactosyltransferase 1 (AY809881), glycoprotein 3-alpha-L-fucosyltransferase A (FN317387), alpha-1,3-mannosyl-glycoprotein 2-beta-N-acetylglucosaminyltransferase (AY812621), and alpha-L-fucosidase-like protein (FN317475) (Additional file 6: Table S5). However, given the inherent complexity of glycosylation and that multiple glycosyltransferases responsible for similar molecular functions are present in the *Schistosoma* genomes [81, 82], it is difficult to conclude that the global level of glycosylation or the expression of specific glycans is higher in adults than in the other stages examined here.

### Genes enriched in eggs

Globally, genes associated with the egg stage are involved in a diversity of biological functions, which may be the result of using samples for analysis that comprise a mixture of immature and mature eggs. In addition to anticipated genes encoding egg proteins, immunogenic miracidial antigens and major egg antigens, a number of genes involved in the cell cycle and proliferation, including meiosis expressed protein 1 (FN317540), meiosis-specific nuclear structural protein 1 (AY810474), mitogen-activated protein kinase 15 (FN317209), putative chromosome segregation protein SMC (AY812773), different isoforms of leishmanolysin-like peptidase (AY811259, FN317512, AY810562 and FN319863) and probably protein VHS3 (FN330961), placenta-specific gene 8 protein (FN317134), placental protein 25 homolog (FN317187) and centrosomal protein of 162 kDa (AY810094), were upregulated in eggs (Additional file 7: Table S6). These transcripts may be enriched in immature eggs, hinting that active cell division is essential for embryonic development.

Further, a group of transcripts encoding tubulin and microtubule-associated motor proteins, i.e. tubulin alpha (FN317215), tubulin beta (FN320386), tubulin beta-2C chain (FN320061), cytoplasmic dynein light chain 1 (FN317588) and 2 (AY914882), dynein light chain 1, axonemal (FN317727), inner dynein arm light chain, axonemal (FN317915), outer dynein arm protein 1 (AY813443), dynein heavy chain 5, axonemal (AY810177), as well as the ciliary and flagellar microtubule components, i.e. tektins (AY814061, AY914954, FN317819 and FN314465), dynein intermediate chain 3 (AY810742) and outer dense fibre protein 3-B (FN318315) were over-expressed in eggs (Additional file 7: Table S6). These transcriptional differences may reflect the fact that a miracidium is enclosed in the eggshell of the mature egg, and once the egg is released into the external environment and contacts freshwater, a high level of movement is required for the larva to hatch and escape from the eggs [83], and to seek the snail intermediate host in order to establish an infection.

Though the miracidium is enclosed by an eggshell, an active parasite-host interplay takes place *via* pores in the egg [83]. On one hand, nutrients (e.g. iron, amino acid and lipid) are acquired by eggs from the host, a process supported by the upregulation of genes involved in transport and exchange activities, such as putative sodium-dependent transporter (FN318875), sodium/hydrogen exchanger (AY815720), sodium/calcium exchanger (FN318247), large neutral amino acids transporter small subunit 2 (FN327074), Y + L amino acid transporter 2 (FN313722), high-affinity choline transporter 1 (FN317430), iron channels (i.e. voltage-gated hydrogen channel (FN318209), two pore calcium channel protein 2 (FN326741), and TWiK family of potassium channels protein (AY813707), and lipid metabolism (i.e. fatty acid-binding protein (FN318753) (Additional file 7: Table S6). On the other hand, it has been shown that major egg products from *S. mansoni* such as ribonuclease omega-1, kappa 5 (FN329842) and IPSE/alpha-1 are released into host tissues and modulate host immune responses [84–87]. In this study, *S. japonicum* homologues of ribonuclease omega-1 (FN330952) and kappa 5 (FN321248) were also enriched in the egg stage, although as yet, no homologue of IPSE/alpha-1 has been identified in this schistosome species.

## Conclusions

In this study, we present the most comprehensive transcriptomic profile to date of four stage-associated genes in *S. japonicum* based on a next-generation DNA chip. The study has revealed the key biological and physiological features of the four development stages: cercariae, hepatic schistosomula, adult parasites and eggs. Overall, this study adds new insights on the developmental biology of *S. japonicum* which further the discovery of novel intervention targets against this persistent parasite and the disease it causes.

## Additional files

Additional file 1: Figure S1. Denaturing agarose gel electrophoresis of RNA samples isolated from different developmental stages (1, cercariae; 2, hepatic schistosomula; 3, adult worms; 4, eggs); one of three biological replicates for each stage are presented. (TIF 82 kb)
Additional file 2: Table S1. List of primer pairs used for qPCR validation. (XLSX 10 kb)
Additional file 3: Table S2. Retrieval of *S. japonicum* stage-enriched genes from the NCBI database based on the DNA chip results. (XLSX 9 kb)
Additional file 4: Table S3. Information on mRNA transcripts enriched in cercariae (forward probe). (XLSX 188 kb)
Additional file 5: Table S4. Information on mRNA transcripts enriched in hepatic schistosomula (forward probe). (XLSX 64 kb)
Additional file 6: Table S5. Information on mRNA transcripts enriched in mixed adult worms (forward probe). (XLSX 150 kb)
Additional file 7: Table S6. Information on mRNA transcripts enriched in eggs (forward probe). (XLSX 287 kb)
Additional file 8: Table S7. Information on EST transcripts enriched in cercariae (forward probe). (XLSX 81 kb)
Additional file 9: Table S8. Information on EST transcripts enriched in hepatic schistosomula (forward probe). (XLSX 25 kb)
Additional file 10: Table S9. Information on EST transcripts enriched in mixed adult worms (forward probe). (XLSX 51 kb)
Additional file 11: Table S10. Information on EST transcripts enriched in eggs (forward probe). (XLSX 51 kb)
Additional file 12: Figure S2. Heatmap for EST transcripts enriched in cercariae, hepatic schistosomula, adult worms and eggs. The heatmap was created by HemI 1.0 based on the transformed data of log_2_ FC value. The data are based on the mean of weighted signal intensity value of forward probe sets (three biological replicates). (TIF 110 kb)
Additional file 13: Table S11. Detailed GO annotation for mRNA transcripts enriched in cercariae. (XLSX 26 kb)
Additional file 14: Table S12. Detailed GO annotation for mRNA transcripts enriched in hepatic schistosomula. (XLSX 18 kb)
Additional file 15: Table S13. Detailed GO annotation for mRNA transcripts enriched in mixed adult worms. (XLSX 22 kb)
Additional file 16: Table S14. Detailed GO annotation for mRNA transcripts enriched in eggs. (XLSX 24 kb)

## Abbreviations

CDS: Coding DNA sequences
DALYs: Disability adjusted life years
DGE: Digital gene expression
ERK: Extracellular signal-regulated kinase
ESTs: Expressed sequence tags
FC: Fold-changes
LAPs: Hydrolysis of lysophosphatidic acids
MAPK: Mitogen-activated protein kinase
PKC: Protein kinase C
SAGE: Serial analysis of gene expression
TES: Trematode eggshell synthesis
UTRs: Untranslated regions
VAL: Venom allergen-like
