# Supplementary figures and images for: A next-generation microarray further reveals stage-enriched gene expression pattern in the blood fluke Schistosoma japonicum
Source: Parasit Vectors. 2017 Jan 10;10:19. doi: 10.1186/s13071-016-1947-x (PMC5223471; doi:10.1186/s13071-016-1947-x)

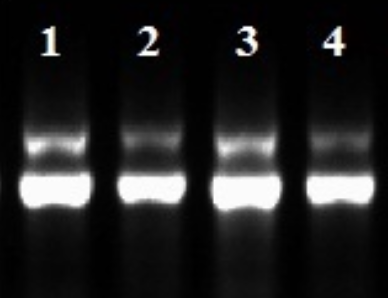

Supplement: Additional file 1: Figure S1. — Denaturing agarose gel electrophoresis of RNA samples isolated from different developmental stages (1, cercariae; 2, hepatic schistosomula; 3, adult worms; 4, eggs); one of three biological replicates for each stage are presented. (TIF 82 kb) [file 13071_2016_1947_MOESM1_ESM.tif]

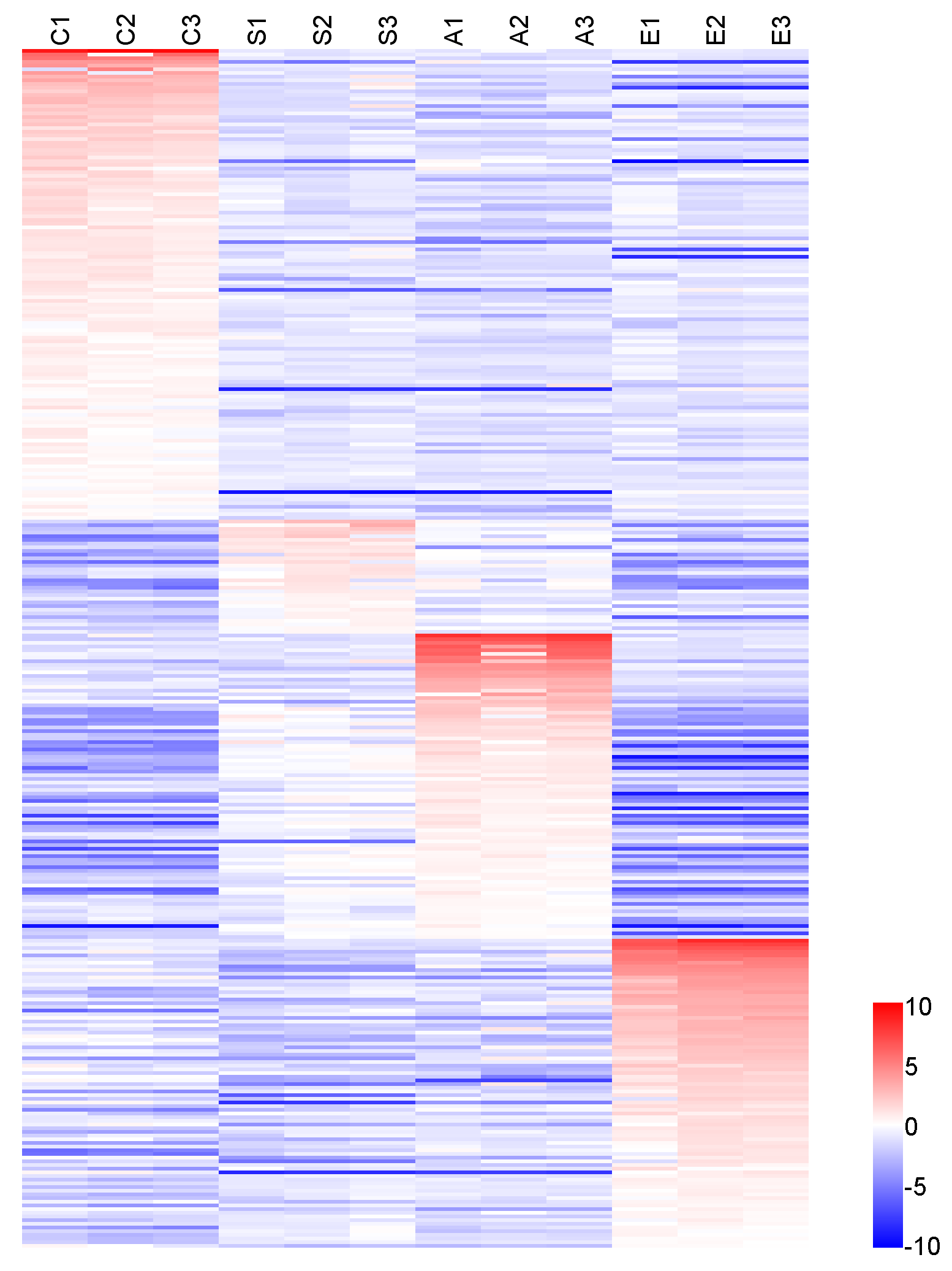

Supplement: Additional file 12: Figure S2. — Heatmap for EST transcripts enriched in cercariae, hepatic schistosomula, adult worms and eggs. The heatmap was created by HemI 1.0 based on the transformed data of log2 FC value. The data are based on the mean of weighted signal intensity value of forward probe sets (three biological replicates). (TIF 110 kb) [file 13071_2016_1947_MOESM12_ESM.tif]
